# HIF1A activates the transcription of lncRNA RAET1K to modulate hypoxia-induced glycolysis in hepatocellular carcinoma cells via miR-100-5p

**DOI:** 10.1038/s41419-020-2366-7

**Published:** 2020-03-09

**Authors:** Yufan Zhou, Yun Huang, Kuan Hu, Zeyu Zhang, Jiajin Yang, Zhiming Wang

**Affiliations:** Department of Hepatobiliary Surgery, Xiangya Hospital, Central South University, Changsha, 410008 China

**Keywords:** Cell biology, Molecular biology

## Abstract

Hepatocellular carcinoma (HCC) remains the primary cause of cancer-related death. Metabolic change is the major characteristic of cancer. The present study attempted to investigate the regulatory mechanisms of HCC energy metabolism from the perspective of noncoding RNA regulation of HIF1A and LDHA. The expression of miR-100-5p expression was significantly suppressed in HCC tissue samples and HCC cell lines under 1% O_2_-induced hypoxia. miR-100-5p overexpression significantly suppressed hypoxia-induced increases in lactate concentration and glucose uptake. Exposure to 1% O_2_ induced HIF1A protein and reduced miR-100-5p expression, while HIF1A silencing dramatically rescued miR-100-5p expression upon 1% O_2_ exposure. In addition, 1% O_2_-induced increases in lactate concentration and glucose uptake were also suppressed by HIF1A silencing. Next, by analyzing available data in TCGA, we found that lncRNA RAET1K was correlated with HIF1A and miR-100-5p.LncRNA RAET1K could downregulate the expression of miR-100-5p by acting as a sponge, while HIF1A bound the lncRNA RAET1K promoter region to activate its transcription. LncRNA RAET1K silencing significantly suppressed HCC cell proliferation and invasion and also suppressed hypoxia-induced increases in lactate concentration and glucose uptake, while miR-100-5p inhibition reversed the effects of lncRNA RAET1K silencing on hypoxia-induced glycolysis in HCC cells. Finally, the expression of HIF1A, lncRNA RAET1K, and LDHA was upregulated in HCC tissue specimens; the expression of miR-100-5p was negatively related to HIF1A, lncRNA RAET1K, and LDHA; and HIF1A, lncRNA RAET1K, and LDHA were positively correlated with each other. In conclusion, the HIF1A/lncRNA RAET1K/miR-100-5p axis modulates hypoxia-induced glycolysis in HCC cells and might affect HCC progression.

## Introduction

Globally, hepatocellular carcinoma (HCC) is not only the most common type of digestive system tumor but also the primary cause of death from cancer^1^. The 5-year survival rate of patients with HCC remains low due to late diagnosis, intrahepatic metastasis, and recurrence after operation^2^. It is urgent to fully understand the molecular mechanisms of HCC development.

One of the characteristic features of tumor cells is metabolic change^3^. Instead of depending on mitochondrial oxidative phosphorylation, cancer cells are more likely to produce energy via glycolysis. This phenomenon, known as the Warburg effect, usually leads to increased in glucose uptake, ATP accumulation, and production of lactic acid in cancer cells^4^. Normally differentiated hepatocytes cannot produce energy through aerobic glycolysis in the non-anaerobic environment. However, in HCC, the metabolic pathways are extensively reprogrammed^5,6^. Tumor cells prefer hypoxic environments and use glycolysis to produce ATP^4,7^. Therefore, focusing on HCC metabolism, especially under low oxygen conditions, may contribute to the treatment of HCC. A a subunit of the heterodimeric transcription factor hypoxia-inducible factor-1 (HIF1), HIF1A is expressed in several human malignancies^8,9^. More importantly, lactate dehydrogenase A (LDHA) is not only a member of the tetrameric enzyme lactate dehydrogenase (LDH) family^10^ and an essential component of the last step of the glycolytic pathway but also exerts critical effects on tumor maintenance and could be regulated by HIF1^11–13^. Therefore, we attempted to investigate the regulatory mechanisms of HCC energy metabolism from the perspective of HIF1A and LDHA regulation.

Recently, emerging evidence has indicated the importance of noncoding RNAs, such as long noncoding RNAs (more than 200 nucleotides in length)^14^ and micro RNAs (miRNAs), which are evolutionally conserved, small noncoding RNAs that could inhibit translation and/or induce mRNA degradation to modulate gene expression at the posttranscriptional level^15^. Noncoding RNAs participate in the modulation of various biological processes of cancer, including glucose metabolism^16^, via a well-established mechanism by which lncRNAs regulate miRNAs by serving as competing endogenous RNAs (ceRNAs)^17,18^. Among deregulated miRNAs in HCC, miR-100-5p was shown to be downregulated in HCCs of humans and rats. The downregulation of miR-100-5p is an initiating event in HCC, and miR-100-5p downregulation is not only related to all phases of HCC^19^, but is also associated with tumor development and poor prognosis of HCC patients^20^. More importantly, miR-100-5p expression has been reported to be downregulated by long-term hypoxia in clear cell renal cell carcinoma^21^. In non-muscle invasive bladder cancer, hypoxia suppressed miR-100-5p expression to induce FGFR3 expression, therefore promoting cell growth^22^. Even so, the function and mechanism by which miR-100-5p exerts its biological effects on glycolysis in HCC in a hypoxic microenvironment remain unclear.

The study first examined miR-100-5p expression in HCC tissue samples and cell lines under hypoxia (1% O_2_), as well as its effect on lactate concentration and glucose uptake under hypoxia. We also detected how HIF1A affected the expression of miR-100-5p under hypoxia. Next, we analyzed the available data in the TCGA database to identify lncRNAs that might be related to both HIF1A and miR-100-5p, and lncRNA RAET1K (isoform 202) was selected. The predicted binding between HIF1A and lncRNA RAET1K, and between lncRNA RAET1K and miR-100-5p was validated. Furthermore, the effect of lncRNA RAET1K on the proliferation and invasion of HCC cells, and the dynamic effects of lncRNA RAET1K and miR-100-5p on glycolysis were examined under hypoxia. Finally, the expression of HIF1A, lncRNA RAET1K, and LDHA in tissue samples was determined, and the correlations of HIF1A, lncRNA RAET1K, LDHA, and miR-100-5p were analyzed. In summary, this study demonstrated the role and the mechanism of the HIF1A/lncRNA RAET1K/miR-100-5p axis in the regulation of HCC glycolysis under hypoxia.

## Materials and methods

### Clinical tissue specimens

A total of 66 paired HCC and adjacent normal tissues were collected from patients (with clear pathological diagnoses) who underwent primary surgical resection at the Xiangya Hospital of Central South University (Changsha, China) with approval by the Research Ethics Committee of the Xiangya Hospital of Central South University (Changsha, China), and written informed consent was obtained from all patients.

### Cell lines, cell culture, and cell transfection

The L02 cell line was obtained from China Center for Type Culture Collection (CCTCC; Wuhan, China) and cultured in the MEM-EBSS: Minimum Essential Medium (MEM with Earle’s Balanced Salts) supplemented with 10% FBS (Invitrogen, Waltham, MA, USA). The HCCLM3 (Cat. CL-0278) cell lines were obtained from ProCell (Wuhan, China) and cultured in RPMI-1640 medium supplemented with 10% FBS (Invitrogen). HepG2 cell line was obtained from ATCC (ATCC® HB-8065™, Manassas, VA, USA) and cultured in MEM (ATCC) supplemented with 10% FBS (Invitrogen). The huh7 cell line was obtained from CCTCC (Cat. GDC0134) and cultured in RPMI-1640 medium supplemented with 10% FBS (Invitrogen). The Hep3B cell line was obtained from ATCC (ATCC® HB-8064™) and cultured in MEM (ATCC) supplemented with 10% FBS (Invitrogen). Cells were cultured in 5% CO_2_ at 37 °C. For hypoxia treatment, cells were exposed to 1% O_2_ for 12 h and then switched to normoxia for an additional 24 h using a tri-gas incubator (SANYO, Japan). Then, cells were harvested for further experiments.

The miR-100-5p mimics, miR-100-5p inhibitor, as well as their negative controls (mimics-NC and inhibitor-NC), were purchased from Genepharma (Shanghai, China). Si-HIF1A#1 (HIF1A 1418-1140) (sense 5′- GCUAUUCACCAAAGUUGAATT -3′; antisense 5′- UUCAACUUUGGUGAAUAGCTT -3′), si-HIF1A#2 (HIF1A 2271-2293) (sense 5′- CCAUAUAGAGAUACUCAAATT -3′; antisense 5′- UUUGAGUAUCUCUAUAUGGTT -3′) and a negative control (si-NC; sense: 5′-UUC UCC GAA CGU GUC ACG UTT -3′; antisense: 5′-ACG UGA CAC GUU CGG AGA ATT -3′) were also purchased from Genepharma (A01003, Shanghai, China). The lncRNA RAET1K smart silencer (a pool containing three siRNAs and three antisense oligonucleotides) and its negative control, lncRNA smart silencer NC, were purchased from RiboBio (siBDM1999A, Guangzhou, China). For RAET1K overexpression, the whole length of RAET1K fragment was cloned into the pcDNA3.1 expression vector by PCR. The primers and siRNA sequences are listed in Table S1. All transfections were performed by using Lipofectamine 3000 reagent (Invitrogen, CA, USA) according to the manufacturer’s instructions.

### RNA fluorescence in situ hybridization

A Specific FAM-labeled lncRNA RAET1K probe and FITC-labeled miR-100-5p probe were obtained from RiboBio (Guangzhou, China). Cells were fixed in 4% formaldehyde for 30 min and then washed with PBS. After permeabilization, cells were prehybridized with hybridization solution and then incubated with both the lncRNA and miRNA probes in hybridization buffer. Cell nuclei were stained with DAPI for 5 min at room temperature. Fluorescence images were obtained by fluorescence microscopy (Olympus, Japan). The probe sequences were listed in Table S1.

### Xenograft nude mice

AntagomiR-100-5p, antagomiR-NC, shRNA negative control vector (lv-sh-NC), and sh-lncRNA RAET1K (sh-RAET1K) vector were obtained from GENECHEM (Shanghai, China). AntagomiR-100-5p, antagomiR-NC sh- RAET1K or sh-NC was transfected into HCCLM3 cells with Lipofectamine 2000. Forty-eight hours later, the transfected cells were harvested for subcutaneous injection. Six-week-old female nude mice (BALB/c-nu/nu) weighing 16–20 g were obtained from the Xiangya Experimental Animal Center (Changsha, China). All experimental procedures were approved by the Ethic Committee of Xiangya Hospital of Central South University. Mice were randomly divided into 4 groups: Control (no transfection) group, sh-NC + antagomiR-NC group, sh-RAET1K + antagomir-NC group and sh-RAET1K + antagomiR-100-5p group. A total of 1 × 10^6^ transfected HCCLM3 cells were suspended in 100 μl of PBS and subcutaneously injected into the left anterior armpits of the nude mice. Twenty-eight days later, the nude mice were sacrificed. The tumor weight and volume were measured. The expression of LDHA was measured by immunohistochemistry (IHC).

### RNA isolation and qRT-PCR

RNA isolation and qRT-PCR were performed as described previously^23^ using a TaqMan MicroRNA Reverse Transcription Kit (4366596, ThermoFisher, CA, USA) or a PrimeScript reverse transcriptase reagent kit (Takara, Osaka, Japan). U6 or Tubulin was used as an internal control. The expression levels were normalized to U6 or Tubulin to produce a 2^−ΔΔCt^ value for the relative expression level. The primers are listed in Table S1.

### Immunoblotting analyses

Immunoblotting analyses were performed to examine protein levels. We separated proteins by SDS–PAGE and transferred proteins to PVDF membranes. The experimental details was performed similarly to previously reported^24,25^. The following primary antibodies were used: anti-LDHA (ab101562, Abcam, Cambridge, MA, USA), anti-HIF1A (ab51608, Abcam), anti-GAPDH (ab181602, Abcam) and anti-β-actin (ab8226, Abcam).

### Immunohistochemical staining

The tumor tissues and xenograft tumor tissues were paraffin-embedded and cut into 3-μm sections and mounted on glass slides for staining with immunoperoxidase, and IHC was performed as previously described^24,25^. The following antibodies were used: anti-LDHA (ab101562, Abcam) and anti-HIF1A (ab51608, Abcam). The pictures were visual under a microscope (Olympus, Japan).

### MTT assay

Cell viability was determined following methods described previously using a cell proliferation kit (C0009, Beyotime, China)^26^. The OD value was measured at 490 nm. The viability of the nontreated cells (control) was defined as 100%, and the viability of cells was calculated based on that of the control group.

### DNA synthesis determination by a 5-bromo-2-deoxyUridine incorporation assay

DNA synthesis in proliferating cells was determined by measuring 5-bromo-2-deoxyUridine (BrdU) incorporation following methods described previously^27^ with a peroxidase-coupled anti-BrdU-antibody (MAB3510P, Sigma-Aldrich). The absorbance values were measured at 450 nm. The background BrdU OD value was determined in cells not exposed to BrdU but stained with the BrdU antibody.

### Luciferase reporter assay

The fragment of lncRNA RAET1K or LDHA-3′UTR was amplified and cloned downstream of Renilla in the psiCHECK2 vector (C8021, Promega, Madison, WI, USA), and the construct was named wt-RAET1K or wt-LDHA. To generate the lncRNA RAET1K or LDHA mutant reporter, we mutated the seed region of lncRNA RAET1K or LDHA-3′UTR to remove the complementarity to miR-100-5p and named the construct mut-RAET1K or mut-LDHA. HEK293 cells (ATCC, USA) were seeded into a 24-well plate and cotransfected with the indicated vectors, and miR-100-5p mimics or miR-100-5p inhibitor. Luciferase assays were performed 48 h after transfection using the Dual-Luciferase Reporter Assay System (Promega). Renilla luciferase activity was normalized to firefly luciferase activity for each transfected well.

To confirm the binding between HIF1A and lncRNA RAET1K, we cotransfected the cells with wt-RAET1K or mut-RAET1K, a pGL3 luciferase reporter construct harboring the HIF1A response element (HIF1A-RE) target sequence and si-NC or si-HIF1A. After 24 h, the activities of firefly luciferase and Renilla luciferase were measured in the cell lysates using the Dual-Luciferase Assay System (Promega, WI, USA).

### Chromatin immunoprecipitation

Cells were transfected or treated, cross-linked by 1% formaldehyde, and DNA was sheared to an average fragment of 400 bp, and immunoprecipitated using antibody against HIF1A (anti-HIF1A, ab1, Abcam). The chromatin immunoprecipitation (ChIP)-PCR primers were designed to amplify the promoter regions containing putative HIF1A binding sites within lncRNA RAET1K. A positive control antibody (against RNA polymerase II) and a negative control nonimmune IgG were used to demonstrate the efficacy of the kit reagents (P-2025-48, Epigentek Group Inc., NY, USA). The immunoprecipitated DNA was subsequently cleaned, and eluted. Specific PCR primers were used to determine the level of VEGF and RAET1K promoter fragments in the immunoprecipitated DNA. The fold-enrichment (FE) was calculated as the ratio of the amplification efficiency of the ChIP sample to that of the nonimmune IgG. The amplification efficiency of RNA Polymerase II was used as a positive control. FE% = 2 (IgG CT-Sample CT) × 100%. The primer sequences were listed in Table S1.

### Measurement of glucose and lactate

Glucose levels were determined using a commercial glucose assay kit, Glucose Colorimetric assay kit II (K686-100, Bio Vision, Milpitas, CA, USA). Glucose uptake was calculated by deducting the detected glucose concentration in the medium from the original glucose concentration. Lactate levels were determined using a lactate colorimetric assay kit (K627-100, Biovision, Milpitas, CA, USA) in accordance with the manufacturer’s instructions. Considering that the cell number in every sample may be different, all the concentrations of glucose or lactate production were finally normalized to the total cell protein concentration. The protein concentration was measured by the BCA protein assay (Cat. 23225, Thermo Fisher Scientific, Waltham, MA, USA).

### Statistical analysis

All data from three independent experiments were expressed as mean ± SD and processed using SPSS17.0 statistical software. The differences between two groups were estimated by Student’s *t*-test; the differences among more than two groups were estimated by one-way ANOVA. A *P* value of <0.05 was considered to be statistically significant.

## Results

### Hypoxic environment promotes glycolysis by inhibiting miR-100-5p and promoting LDHA expression

**Fig. 1:**
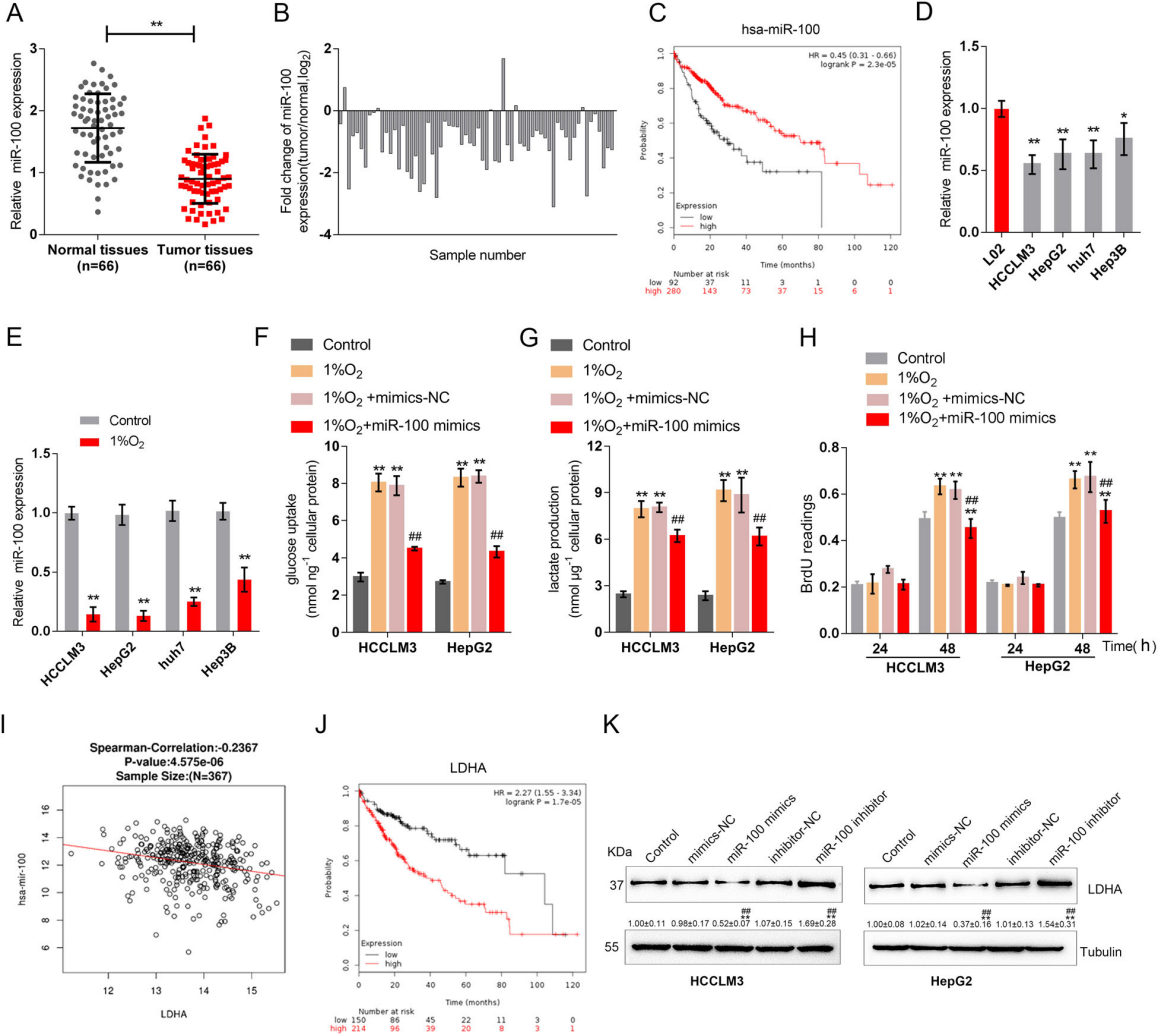
Hypoxia promotes glycolysis by inhibiting miR-100-5p and promoting LDHA expression. **a** The expression of miR-100-5p in 66 paired hepatocellular carcinoma (HCC) and adjacent nontumor tissues was determined by real-time PCR. (*n* = 66). **b** The fold change of miR-100-5p in tissue samples represented as tumor/normal, log_2_ (*n* = 66). **c** Kaplan–Meier overall survival curves for patients with HCC categorized according to relative miR-100-5p expression level. Data based on TCGA database. **d** The expression of miR-100-5p was determined in L02, HCCLM3, HepG2, Huh7, and Hep3B cells by real-time PCR (*n* = 5). **e** HCCLM3, HepG2, huh7, and Hep3B cells were exposed or not exposed to 1% O_2_ and examined for the expression of miR-100-5p (*n* = 5). **f**–**h** HCCLM3 and HepG2 cells were transfected with miR-100-5p mimics and exposed to 1% O_2_ and examined for lactate concentration, glucose uptake, and cell viability by BrdU assays (*n* = 3). **i** The expression of miR-100-5p was negatively correlated to LDHA according to the TCGA database. **j** Kaplan–Meier overall survival curves for patients with HCC categorized according to relative LDHA expression level. **k** HCCLM3 and HepG2 cells were transfected with miR-100-5p mimics or miR-100-5p inhibitor and examined for the protein levels of LDHA. *n* = 3. *P < 0.05, **P < 0.01, compared to control group; ^#^*P* < 0.05, ^##^*P* < 0.01, compared to 1% O_2_ + mimics-NC group.

To confirm that miR-100-5p participates in the regulation of glycolysis, the study first examined miR-100-5p expression in tissue samples or in response to hypoxic conditions. The expression of miR-100-5p in HCC tissue samples was significantly downregulated, compared to that in noncancerous tissues (*n* = 66, Fig. 1a). The fold change of miR-100-5p was represented as log_2_(tumor/normal) in Fig. 1b. Data from the TCGA online database showed that a higher miR-100-5p expression was correlated with higher survival probability (Fig. 1c).

Next, the expression of miR-100-5p was determined in four HCC cell lines, HCCLM3, Huh7, Hep3B, and HepG2, and the normal cell line L02. As shown in Fig. 1d, miR-100-5p expression was significantly downregulated in all four HCC cell lines compared to L02 cells. Then, HCCLM3, Huh7, Hep3B, and HepG2 cells were exposed to 1% O_2_ to mimic hypoxic conditions, and miR-100-5p expression was examined. As shown in Fig. 1e, miR-100-5p expression was significantly suppressed by hypoxia in all four HCC cell lines and was the most suppressed in HCCLM3 and HepG2 cells; thus, HCCLM3 and HepG2 cells were used in further analyses. We modified miR-100-5p expression by transfecting HCCLM3 and HepG2 cells with miR-100-5p mimics/miR-100-5p inhibitor, and performed real-time PCR to verify the transfection efficiency (Fig. S1A). miR-100-5p-overexpressing HCCLM3 and HepG2 cells were then examined for lactate concentration and glucose uptake under normoxia or hypoxic condition. The lactate concentration and glucose uptake were significantly induced by hypoxia but inhibited by miR-100-5p overexpression (Fig. 1f, g), suggesting that miR-100-5p overexpression might block glycolysis in cancer cells in a hypoxic microenvironment. Moreover, BrdU assays performed under the same conditions indicated that tumor cell viability was promoted by hypoxia but significantly suppressed by miR-100-5p overexpression (Fig. 1h). These data suggest that miR-100-5p might modulate HCC cell phenotypes by affecting glycolysis under hypoxia.

More importantly, there was a negative association between miR-100-5p expression and LDHA expression (Fig. 1i). As a member of the LDH family of tetrameric enzymes^10^, LDHA is not only a vital component of the last step of the glycolytic pathway but also exerts significant effects on tumor maintenance, according to the TCGA online database. Additionally, as shown by the TCGA analysis, higher LDHA expression was correlated with lower survival probability (Fig. 1j). Additionally, in HCCLM3 and HepG2 cells, miR-100-5p overexpression downregulated, while miR-100-5p inhibition upregulated LDHA protein levels (Fig. 1k). To further confirm the negative correlation between miR-100-5p and LDHA, a luciferase reporter assay was performed by constructing two different LDHA 3′UTR luciferase reporter vectors, wild-type (wt-LDHA 3′UTR) and mutant-type (mut-LDHA 3′UTR) (Fig. S1B). These vectors were cotransfected into 293T cells with miR-100-5p mimics or miR-100-5p inhibitor, and the luciferase activity was determined. As shown in Fig. S1C, the luciferase activity of wt-LDHA 3′UTR was significantly suppressed by miR-100-5p overexpression but increased by miR-100-5p inhibition. In addition, after mutating the predicted miR-100-5p binding site, the changes in luciferase activity were abolished (Fig. S1C). Moreover, as shown in Fig. S2, miR-100-5p overexpression significantly decreased while miR-100-5p inhibition increased lactate concentration and glucose uptake in HCC cells. In summary, miR-100-5p exerts its biological effect on glycolysis in HCC cells under hypoxic conditions (1% O_2_).

### HIF1A reverses the effect of 1% O_2_-induced hypoxia on miR-100-5p expression and glycolysis

**Fig. 2:**
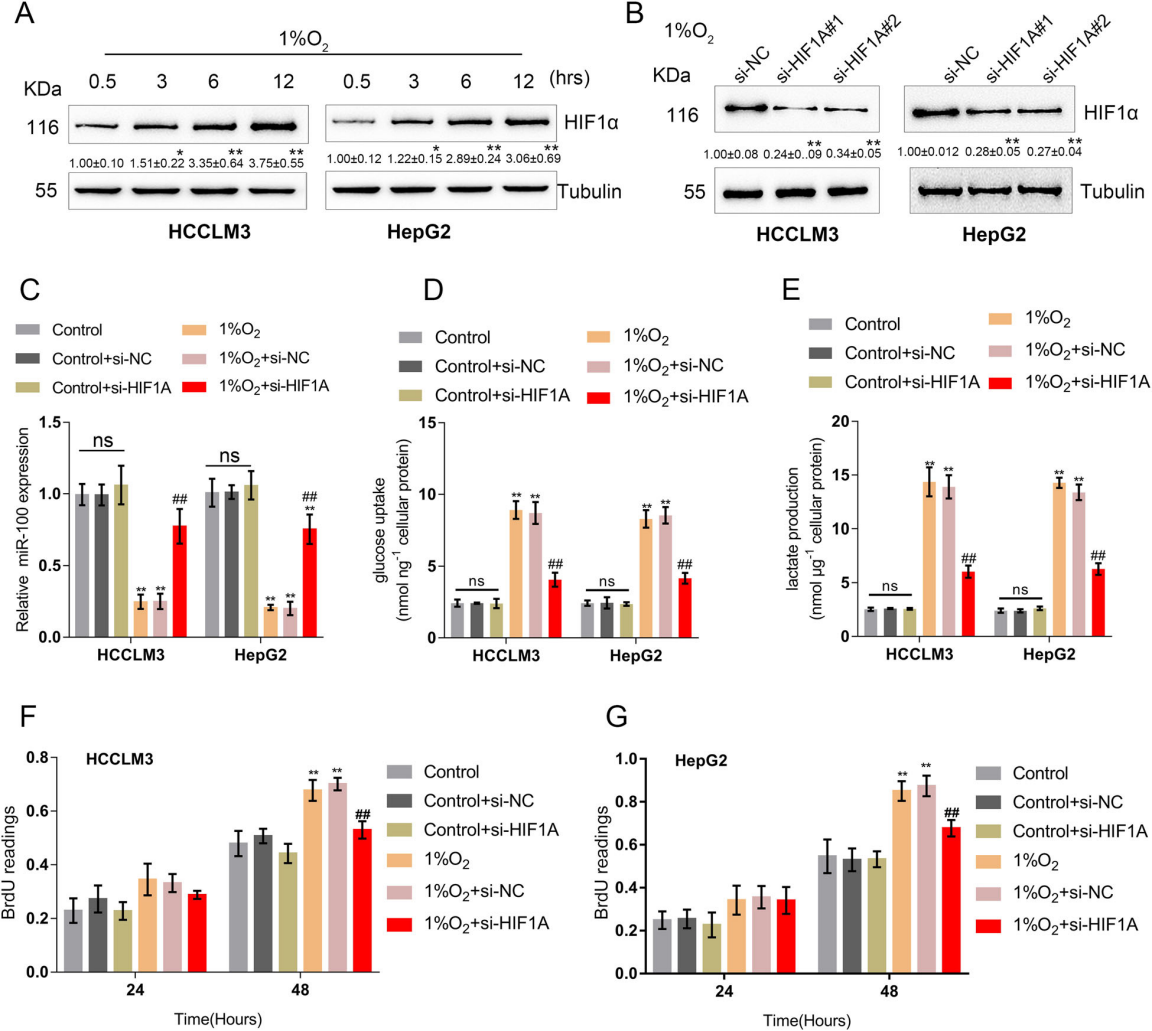
HIF1A reverses the effect of 1% O_2_-induced hypoxia on miR-100-5p expression and glycolysis. **a** HCCLM3 and HepG2 cells were exposed to 1% O_2_ for 0.5, 3, 6, or 12 h and examined for the protein levels of HIF1A (*n* = 3). **b** HIF1A silencing in HCCLM3 and HepG2 cells was achieved by transfection with si-HIF1A#1 or si-HIF1A#2 under 1% O_2_ exposure, as confirmed by Immunoblotting (*n* = 3.). HCCLM3 and HepG2 cells were transfected with si-HIF1A, exposed to 1% O_2_ and examined for **c** the expression of miR-100-5p (*n* = 5), **d**, **e** lactate concentration and glucose uptake, and **f**, **g** the cell viability by BRDU assays. *n* = 3. *P < 0.05, **P < 0.01, compared to control group; ^#^*P* < 0.05, ^##^*P* < 0.01, compared to 1% O_2_ group.

HIF triggers LDHA upregulation, while LDHA and HIF1A form a positive feedback loop leading to an increase in lactic acid concentration^28^. Next, we investigated whether HIF1A could affect the inhibitory effect of hypoxia on miR-100-5p expression. In HCCLM3 and HepG2 cells, the protein levels of HIF1A were significantly induced by 1% O_2_ exposure in a time-dependent manner (Fig. 2a). Under 1% O_2_-induced hypoxic conditions, HIF1A silencing was achieved in HCCLM3 and HepG2 cells by transfection with si-HIF1A, as confirmed by immunoblotting (Fig. 2b). HIF1A overexpression was generated by the transfection of the HIF1A-overexpressing vector, as verifed by immunoblotting (Fig. S3). As shown in Fig. 2c, 1% O_2_-induced suppression of miR-100-5p expression was significantly reversed by HIF1A silencing in both HCCLM3 and HepG2 cell lines. In addition, 1% O_2_-induced increases in lactate concentration and glucose uptake were reduced considerably by HIF1A silencing (Fig. 2d, e), indicating that HIF1A could affect the expression of miR-100-5p, therefore modulating the glycolysis in HCC cells under 1% O_2_-induced hypoxia. Similarly, BrdU assays suggested that 1% O_2_-induced HCC cell proliferation could be significantly suppressed by HIF1A silencing (Fig. 2f, g).

### The HIF1A/lncRNA RAET1K axis regulates miR-100-5p expression

**Fig. 3:**
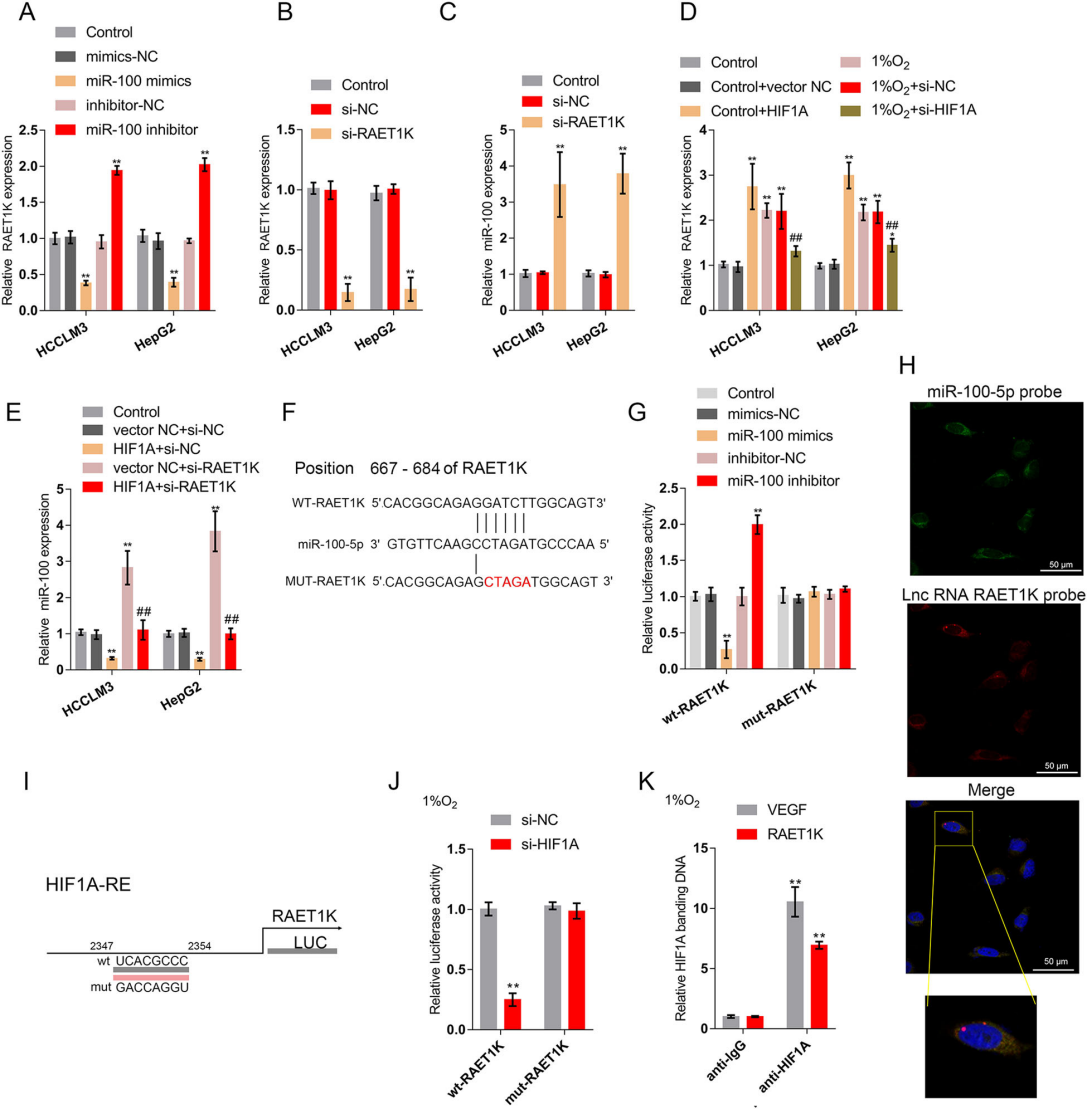
The HIF1A/lncRNA RAET1K axis regulates miR-100-5p expression. **a** HCCLM3 and HepG2 cells were transfected with miR-100-5p mimics or miR-100-5p inhibitor and examined for the expression of lncRNA RAET1K expression (*n* = 5). **b** LncRNA RAET1K silencing was conducted in HCCLM3 and HepG2 cells by transfection with si-RAET1K, as confirmed by real-time PCR (*n* = 5). **c** miR-100-5p expression in response to lncRNA RAET1K silencing was examined by real-time PCR (*n* = 5). **d** HCCLM3 and HepG2 cells were transfected with si-HIF1A or HIF1A-overexpressing vector and exposed to 1% O_2_ or normoxia and examined for the expression of lncRNA RAET1K by real-time PCR (*n* = 5). **e** HCCLM3 and HepG2 cells were cotransfected with HIF1A-overexpressing vector and si-RAET1K and examined for the expression of miR-100-5p. *n* = 5. **f** A schematic diagram showing the structures of the wild-type and mutant-type lncRNA RAET1K luciferase reporter vectors containing the wild-type or mutant-type miR-100-5p binding site. **g** HEK293 cells were cotransfected with the above vectors and miR-100-5p mimics/inhibitor and examined for luciferase activity. *n* = 3. **h** FISH assay of lncRNA RAET1K and miR-100-5p showed that lncRNA RAET1K colocalized with miR-100-5p. **i** A schematic diagram of a potential HIF1A binding element in the promoter region of lncRNA RAET1K predicted by Jaspar database. A wt-RAET1K promoter luciferase reporter vector and a mut-RAET1K promoter luciferase reporter vector were constructed. **j** HEK293 cells were co-transfected with wild-type or mutant-type lncRNA RAET1K and si-HIF1A and examined for the luciferase activity. *n* = 3. **k** ChIP assays were performed using anti-HIF1A to detect the level of HIF1A antibody binding to lncRNA RAET1K and VEGF promoter compared to that of IgG (*n* = 3). *P < 0.05, **P < 0.01, compared to control group; ^#^*P* < 0.05, ^##^*P* < 0.01, compared to 1% O_2_ + si-HIF1A group.

In addition to those of miRNAs, the roles of lncRNAs in glycolysis have been reported^29–31^. LncRNAs could suppress the expression of miRNAs and exert their effect on miRNA functions by acting as miRNA sponges through direct targeting^32^. Next, we analyzed data in TCGA to identify lncRNAs that are negatively correlated with miR-100-5p and positively correlated with HIF1A, and lncRNA RAET1K was selected. Moreover, a higher expression of lncRNA RAET1K was associated with lower survival probability (Fig. S4). Consistent with these online data, miR-100-5p could negatively modulate lncRNA RAET1K expression in HCCLM3 and HepG2 cell lines (Fig. 3a). To investigate the effects of lncRNA RAET1K, we transfected cells with si-RAET1K to silence lncRNA RAET1K, and performed real-time PCR to verify the transfection efficiency (Fig. 3b).The results showed that LncRNA RAET1K silencing could negatively modulate miR-100-5p expression in HCCLM3 and HepG2 cell lines (Fig. 3c), indicating that lncRNA RAET1K could indeed act as a sponge of miR-100-5p. In addition, RAET1K overexpression reduced the miR-100-5p level in HCCLM3 and HepG2 cell lines further confirmed the sponge effect of RAET1K on miR-100-5p (Fig. S5A, B). More importantly, HIF1A overexpression under normoxia significantly increased lncRNA RAET1K expression (Fig. 3d), while the 1% O_2_-induced increase in lncRNA RAET1K expression was considerably inhibited by HIF1A silencing (Fig. 3d), indicating that HIF1A might affect lncRNA RAET1K expression, subsequently modulating miR-100-5p expression under hypoxia. As expected, HIF1A overexpression significantly inhibited, while lncRNA RAET1K silencing promoted the expression of miR-100-5p; lncRNA RAET1K significantly reversed the effect of HIF1A overexpression on miR-100-5p expression (Fig. 3e).

To investigate the molecular mechanism, we performed luciferase reporter assays to examine the putative binding between lncRNA RAET1K and miR-100-5p predicted by an online tool (Fig. 3f). We constructed two lncRNA RAET1K luciferase reporter vectors, wild-type and mutant-type RAET1K. In the putative miR-100-5p binding site of mutant-type RAET1K reporter vector, 5 bases were mutated (Fig. 3f). After cotransfection, the luciferase activity of the wt-RAET1K vector was remarkably downregulated by miR-100-5p overexpression, whereas it was upregulated by miR-100-5p inhibition, and mutating the putative miR-100-5p binding site eliminated the changes in luciferase activity (Fig. 3g). Moreover, the fluorescence in situ hybridization (FISH) results further confirmed the colocation of lncRNA RAET1K and miR-100-5p in the cytoplasm (Fig. 3h). The lncRNA RAET1K promoter region also contained a HIF1A response element (HIF1A RE); thus, the study performed luciferase reporter assays to examine the binding between HIF1A and lncRNA RAET1K by constructing lncRNA RAET1K reporter vectors containing wild-type or mutated HIF1A binding sites (Fig. 3i). We cotransfected these vectors into HEK293 cells along with si-HIF1A under hypoxia. The luciferase activity of wt-RAET1K was significantly suppressed by HIF1A silencing, while mutating the putative HIF1A binding site was eliminated the changes in luciferase activity (Fig. 3j). To provide further evidence on the binding, we used a HIF1A antibody to conduct the ChIP assay under hypoxia. As shown in Fig. 3k, the level of HIF1A antibody binding to HIF1A RE within the lncRNA RAET1K promoter was significantly increased compared to that of IgG and was similar to that of VEGF, a widely-recognized HIF1A target gene^33^. These data indicate that HIF1A directly targets lncRNA RAET1K, while lncRNA RAET1K directly targets miR-100-5p to form a regulatory axis that could modulate glycolysis in HCC cells under hypoxia.

### Effects of lncRNA RAET1K on the proliferation and invasion of HCC cells

**Fig. 4:**
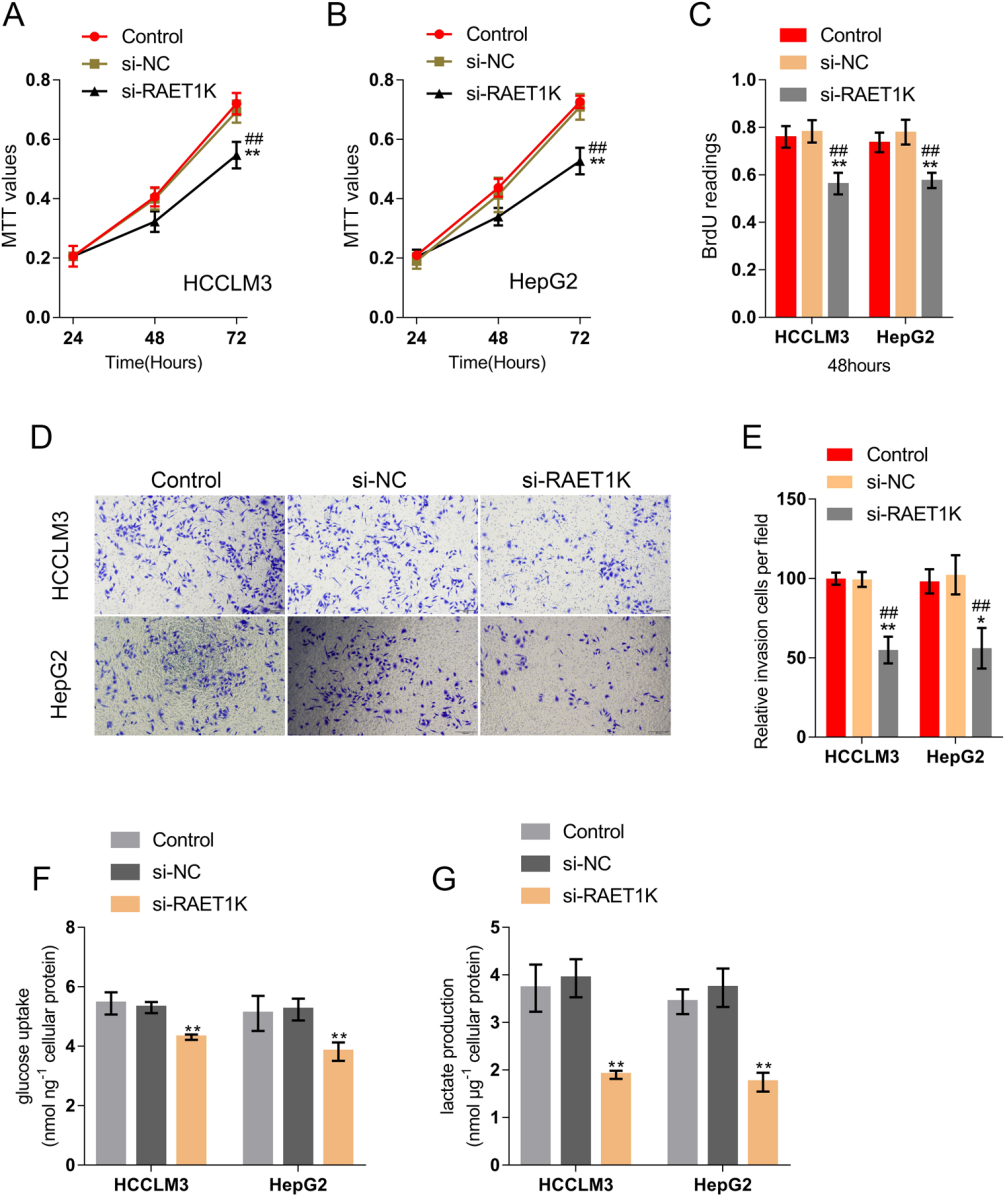
Effects of lncRNA RAET1K on HCC cell proliferation and invasion. HCCLM3 and HepG2 cells were transfected with si-RAET1K under normoxic conditions and examined for **a**, **b** cell viability by MTT assays (*n* = 5), **c** DNA synthesis capacity by BrdU assays (*n* = 3), **d**, **e** cell invasion by Transwell assays, and **f**, **g** the glucose uptake and lactate concentration (*n* = 3). *P < 0.05, **P < 0.01, compared to control group.

Next, we evaluated how lncRNA RAET1K silencing affected the ability of HCC cells to proliferate and invade. According to Fig. 4a–c, lncRNA RAET1K silencing significantly inhibited the growth and DNA synthesis capacity of HCC cell lines. Similarly, lncRNA RAET1K silencing also remarkably inhibited HCC cell invasion (Fig. 4d, e). More importantly, lncRNA RAET1K silencing also significantly decreased glucose uptake and lactate concentration in HCC cells (Fig. 4f, g). In contrast, RAET1K overexpression increased glucose uptake and lactate concentration in HCC cells (Fig. S5C, D). These data indicate that lncRNA RAET1K serves as an oncogene in HCC cells, possibly through a glycolysis-related mechanism.

### The lncRNA RAET1K/miR-100-5p axis modulates glycolysis under hypoxia

**Fig. 5:**
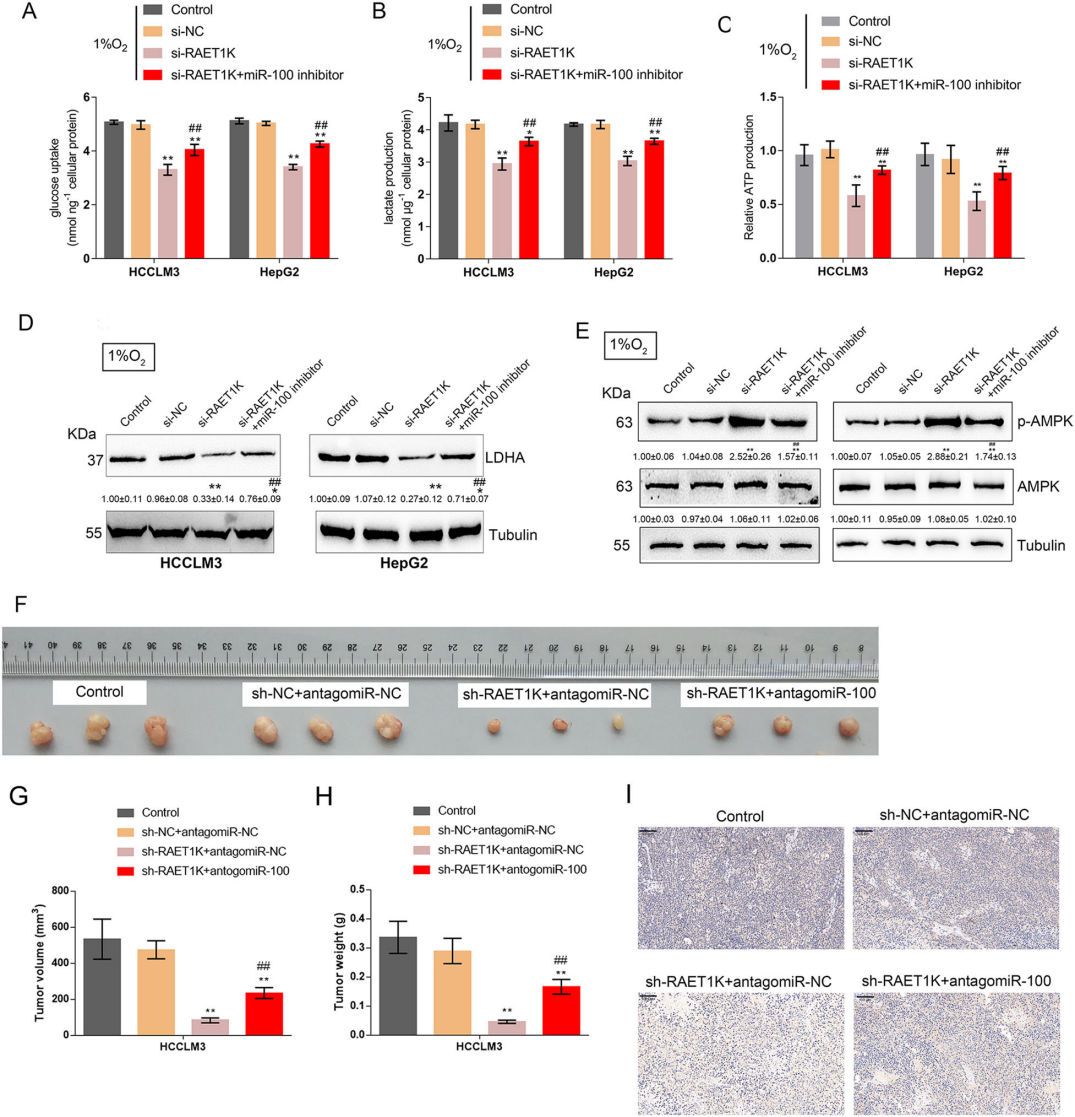
The lncRNA RAET1K/miR-100-5p axis modulates glycolysis under hypoxia. HCCLM3 and HepG2 cells were cotransfected with si-RAET1K and miR-100-5p inhibitor with 1% O2 exposure and examined for **a**, **b** lactate concentration and glucose uptake, **c** ATP production, **d** the protein levels of LDHA, and **e** the protein levels of p-AMPK and AMPK. (*n* = 3). **f** Xenograft nude mice models were performed. HCCLM3 cell xenograft tumor volume (**g**) and tumor weight (**h**), and **i** the IHC staining of LDHA in tumors from the control group, sh-NC + antagomiR-NC group, sh-RAET1K + antagomiR-NC group and sh-RAET1K + antagomiR-100-5p group (*n* = 6). **P < 0.01, compared to control group; ^#^*P* < 0.05, ^##^*P* < 0.01, compared to si-RAET1K group.

We have revealed that HIF1A overexpression could affect miR-100-5p expression and glycolysis in HCC cells, while HIF1A, lncRNA RAET1K, and miR-100-5p could form a regulatory axis. Next, we evaluated the roles of the lncRNA RAET1K/miR-100-5p axis in glycolysis in HCC cells under 1% O_2_-induced hypoxia. We cotransfected HCCLM3 and HepG2 cells with si-RAET1K and miR-100-5p inhibitor under 1% O_2_ exposure and examined lactate concentration, glucose uptake, ATP levels, and LDHA protein were then examined. As previously demonstrated, 1% O_2_-induced hypoxia significantly increased, while lncRNA RAET1K silencing decreased lactate concentration, glucose uptake, and the protein levels of LDHA and increased the phosphorylation of AMPK, while under 1% O_2_-induced hypoxia, miR-100-5p inhibition significantly reversed the effects of lncRNA RAET1K silencing on glycolysis and AMPK phosphorylation in HCC cells (Fig. 5a, b, d, e). Accordingly, lncRNA RAET1K silencing significantly reduced, while miR-100-5p inhibition increased the ATP production; the effects of lncRNA RAET1K silencing were reversed considerably by miR-100-5p inhibition (Fig. 5c). In summary, the lncRNA RAET1K/miR-100-5p axis could modulate glycolysis in HCC cells under 1% O_2_-induced hypoxic conditions. To further validate the effect of LncRNA RAET1K/miR-100-5p axis in vivo, we performed HCCLM3 cell xenograft nude mice model. As Fig. 5f–h shown, lncRNA RAET1K knockdown reduced the tumor growth, but miR-100-5p inhibition by antagomiR-100-5p could partly reverse the reduction (Fig. 5f–h). The IHC staining showed that lncRNA RAETK1K knockdown reduced the LDHA protein expression but miR-100-5p inhibition increased LDHA expression (Fig. 5i).

### Expression and correlation of lncRNA RAET1K, LDHA, HIF1A, and miR-100-5p in tissue samples

**Fig. 6:**
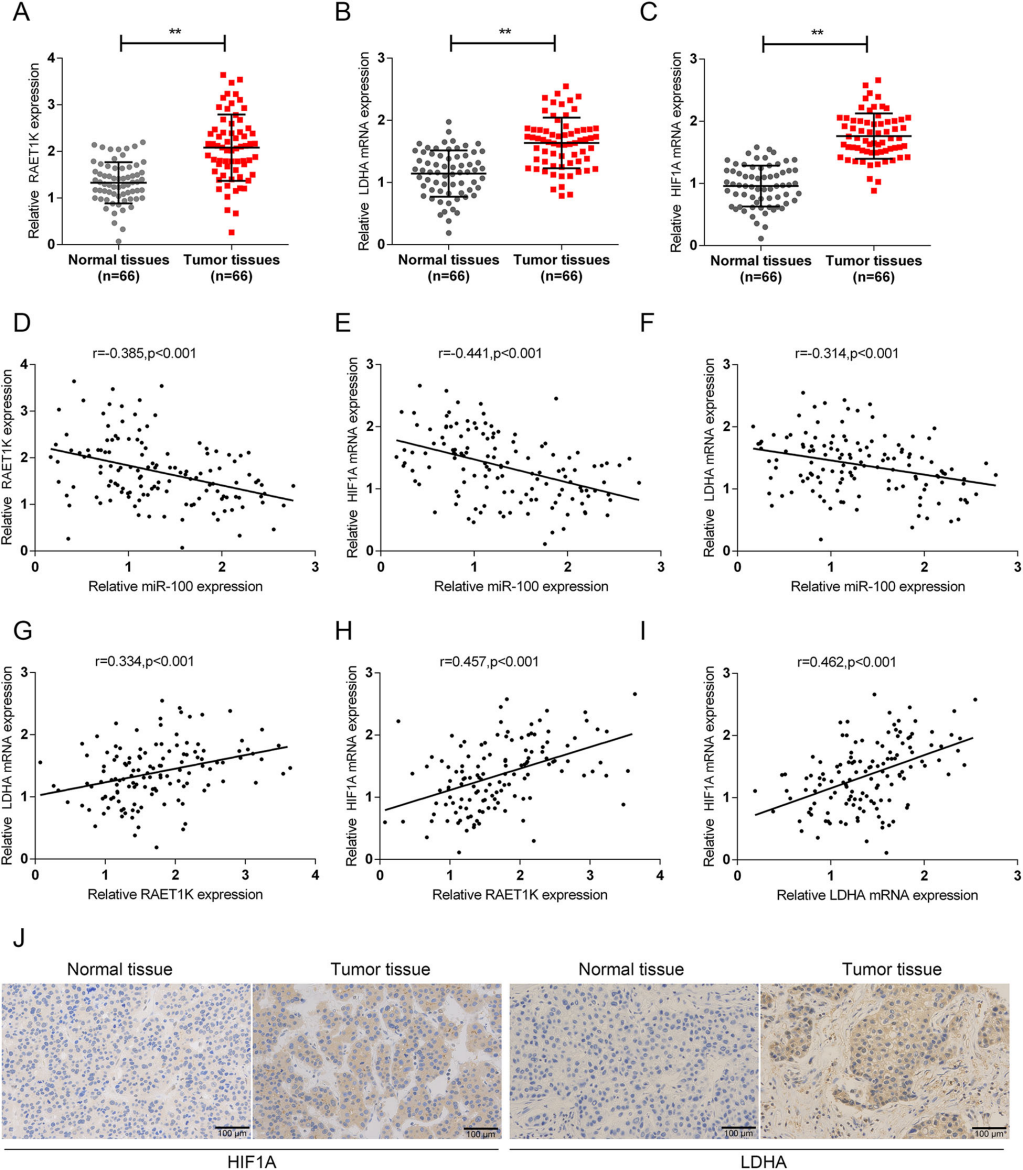
Expression and correlation of lncRNA RAET1K, LDHA, HIF1A, and miR-100-5p in tissue samples. **a**–**c** The expression levels of lncRNA RAET1K, LDHA, and HIF1A in 66 paired HCC and noncancerous tissues were determined by real-time PCR (*n* = 66). **d**–**i** The correlations of lncRNA RAET1K, LDHA, HIF1A, and miR-100-5p in tissue samples were analyzed by Pearson’s correlation analyses. **j** HIF1A and LDHA content and distribution in tissue samples were detected by IHC staining (*n* = 8). **P < 0.01.

As further proof, lncRNA RAET1K, LDHA, and HIF1A expression in tissue samples was examined. According to Fig. 6a–c, the expression of lncRNA RAET1K, LDHA, and HIF1A was significantly upregulated in HCC cells compared with nontumor cells. Furthermore, the expression of miR-100-5p within tissue specimens was negatively related to lncRNA RAET1K, LDHA, and HIF1A (Fig. 6d–f), and lncRNA RAET1K, LDHA, and HIF1A were positively correlated with each other (Fig. 6g–i). The IHC staining results also confirmed that the protein levels of HIF1A and LDHA were higher in tumor tissues, than in normal tissues (Fig. 6j).

## Discussion

**Fig. 7:**
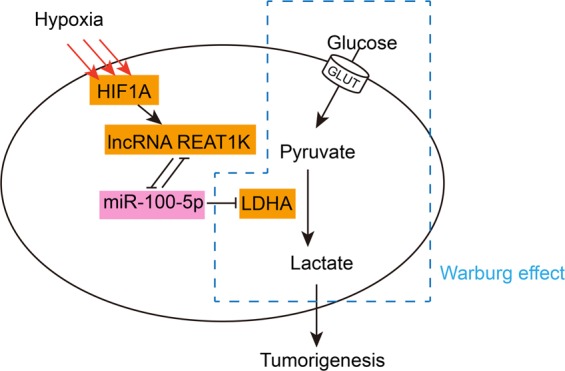
A schematic diagram of the mechanism. The HIF1A/lncRNA RAET1K/miR-100-5p axis modulates hypoxia-induced glycolysis in HCC cells and then might affect HCC progression.

Herein, as shown in Fig. 7, we revealed that the expression of miR-100-5p was significantly suppressed in HCC tissue samples and HCC cell lines under hypoxic conditions (1% O_2_). miR-100-5p overexpression significantly suppressed hypoxia-induced increases in lactate concentration and glucose uptake. Hypoxia-induced HIF1A protein expression and reduced miR-100-5p expression while HIF1A silencing significantly rescued miR-100-5p expression upon hypoxia. In addition, hypoxia-induced increases in lactate concentration and glucose uptake were also suppressed by HIF1A silencing. Next, by analyzing the available data in TCGA, we found that lncRNA RAET1K was correlated with HIF1A and miR-100-5p. LncRNA RAET1K could downregulate the expression of miR-100-5p by acting as a sponge, while HIF1A bound the lncRNA RAET1K promoter region to activate its transcription. LncRNA RAET1K silencing significantly suppressed HCC cell proliferation and invasion, and suppressed hypoxia-induced increases in lactate concentration and glucose uptake, while miR-100-5p inhibition reversed the effect of lncRNA RAET1K silencing on hypoxia-induced glycolysis in HCC cells. Finally, the expression of HIF1A, lncRNA RAET1K, and LDHA was upregulated in HCC tissue specimens; the expression of miR-100-5p was negatively related to HIF1A, lncRNA RAET1K, and LDHA; and HIF1A, lncRNA RAET1K, and LDHA were positively correlated with each other.

Recently, it has been revealed that the dysregulation of miR-100-5p contributes to human malignant tumors, such as HCC. Zhou et al.^34^ showed that suppression of miR-100-5p in HCC tissue samples was remarkably related to venous invasion, advanced TNM stage, an incomplete capsule of tumor nodules, reduced cell differentiation, and shorter survival time without recurrence. Moreover, miR-100-5p significantly inhibited HCC cell migration and invasion in vitro, and xenografts of HCC cells with stable expression of miR-100-5p significantly reduced the lung metastasis in vivo. Regarding the molecular mechanism, miR-100-5p suppression upregulates the ICMT-Rac1 signaling pathway, thus promoting HCC cell metastasis. Another group revealed that miR-100-5p could promote Atg7-dependent autophagy and subsequent apoptotic cell death. Consistent with these findings, miR-100-5p suppressed HCC cell viability in vivo in mouse xenograft model^35^. During last few decades, aerobic glycolysis has been regarded as a cancer hallmark^36^. Elevated lactate levels are strongly negatively correlated with patient outcomes in many cancers^37,38^. Hence, enhanced glucose consumption may play a critical role in cancer progression. Glycolysis-related genes are ubiquitously overexpressed in cancers^39,40^. Herein, it was demonstrated that under hypoxia, miR-100-5p overexpression dramatically suppressed hypoxia-induced increases in lactate concentration and glucose uptake, indicating that miR-100-5p might affect HCC progression by modulating glycolysis in HCC cells, which adds to previous studies demonstrating that miR-100-5p affects HCC cell proliferation, invasion, and migration. More importantly, HIF1A negatively regulated miR-100-5p expression under hypoxic condition; in addition, HIF1A silencing inhibited hypoxia-induced glycolysis in HCC cells, indicating that deregulation of miR-100-5p could be related to HIF1A function in hypoxia-induced glycolysis in HCC cells.

As we have mentioned, HIF1A is increased in various human malignancies^8,9^. Overexpressed HIF1A could be intimately related to unfavorable prognosis in HCC^41^. HIF1A dimerizes with HIF1β and activates the transcription of target genes, which exert the significant effects on cancer cell metabolic reprogramming^42,43^. More importantly, HIF1 regulates the transcription of several critical factors in glycolysis^11–13^. So far, the most widely accepted mechanism by which miRNAs exert their biological functions is through interaction with lncRNAs^44,45^, another type of noncoding RNA. A wide range of previous studies have also indicated that lncRNAs play a critical role in HCC progression^46–48^; however, few studies have investigated the role of lncRNA-mediated glucose metabolism reprogramming in HCC progression^49^. Thus, we attempted to identify lncRNAs that might be related to both HIF1A and miR-100-5p and found that lncRNA RAET1K was positively associated with HIF1A but negatively associated with miR-100-5p. As predicted by online tools, HIF1A directly targets the lncRNA RAET1K promoter to activate its transcription while lncRNA RAET1K directly targets miR-100-5p to suppress its expression. These findings indicate that HIF1A, lncRNA RAET1K, and miR-100-5p could form a regulatory axis to modulate glycolysis in HCC to affect HCC progression.

According to the TCGA database, lncRNA RAET1K expression was upregulated in lung adenocarcinoma; Sui et al.^50^ reported a similar result that lncRNA RAET1K was upregulated in lung adenocarcinoma tumor tissues and that lncRNA RAET1K exhibited a significant prognostic value for lung adenocarcinoma. However, the specific role of lncRNA RAET1K in HCC progression has not yet been investigated. Herein, we found that lncRNA RAET1K silencing dramatically suppressed the ability of HCC cells to proliferate and invade. Regarding the molecular mechanism, lncRNA RAET1K silencing significantly inhibited while miR-100-5p inhibition enhanced hypoxia-induced increases in lactate concentration and glucose uptake; more importantly, miR-100-5p suppression significantly attenuated the effects of lncRNA RAET1K silencing on hypoxia-induced glycolysis in HCC cells, indicating that the lncRNA RAET1K/miR-100-5p axis might affect HCC cell phenotypes by modulating hypoxia-induced glycolysis in HCC cells. As a further confirmation, the expression of HIF1A, lncRNA RAET1K, and LDHA was significantly upregulated in HCC tissues. miR-100-5p expression was negatively related to HIF1A, lncRNA RAET1K, and LDHA whereas HIF1A, lncRNA RAET1K, and LDHA could be positively associated with each other. These findings suggest that the lncRNA RAET1K/miR-100-5p axis might also affect HCC progression, which needs further in vivo investigation.

## Conclusion

The HIF1A/lncRNA RAET1K/miR-100-5p axis modulates hypoxia-induced glycolysis in HCC cells and then might affect HCC progression (Fig. 7).

## Supplementary information

supplementary figures legends

fig.s1

fig.s2

fig.s3

fig.s4

fig.s5

table s1
