# Supplementary material for: HIF1A activates the transcription of lncRNA RAET1K to modulate hypoxia-induced glycolysis in hepatocellular carcinoma cells via miR-100-5p
Source: Cell Death Dis. 2020 Mar 9;11(3):176. doi: 10.1038/s41419-020-2366-7 (PMC7062743; doi:10.1038/s41419-020-2366-7)
Supplement: Supplementary file 1 — supplementary figures legends [file 41419_2020_2366_MOESM1_ESM.docx]

**Fig. S1 Confirmation of the target binding site between miR-100-5p and LDHA** (A) The transfection efficiency of miR-100-5p mimics and miR-100-5p inhibitor in HCCLM3 and HepG2 cells confirmed by real-time PCR (n=5). (B) The predicted miR-100-5p binding site in the LDHA 3'UTR is shown. Wild-type and mutant-type LDHA 3'UTR luciferase reporter vectors were constructed as described in the Materials and methods section and named wt-LDHA 3'UTR and mut-LDHA 3'UTR, respectively. (C) These vectors were cotransfected into 293T cells with miR-100-5p mimics or miR-100-5p inhibitor and the changes in luciferase activity were determined. n=3. ***P*<0.01.

**Fig. S2 Effects of miR-100-5p on HCC cell glycolysis** (A) HCCLM3 and HepG2 cells were transfected with miR-100-5p mimics or miR-100-5p inhibitor and examined for the lactate concentration (A) and glucose uptake (B) (n=3). ***P*<0.01, compared to mimics-NC or inhibitor-NC.

**Fig. S3 The overexpression efficiency of HIF1A.** HCCLM3 and HepG2 cells were transfected with the pcDNA3.1-HIF1A overexpression vector, and 48 h later, HIF1A protein levels were measured by immunobloting (A), the statistic analysis were shown in B (n=3). ***P*<0.01, compared to pcDNA3.1 group.

**Fig. S4 Correlation of miR-100-5p, lncRNA RAET1K, and HIF1A in tissue samples according to the TCGA database** (A) The correlation of lncRNA RAET1K and miR-100-5p. (B) The correlation between lncRNA RAET1K and HIF1A. (C) Kaplan-Meier overall survival curves for patients with HCC classified according to relative lncRNA RAET1K expression level.

**Fig. S5 Effects of RAET1K overexpression on miR-100-5p expression and HCC cell metabolism** (A) LncRNA RAET1K overexpression was achieved in HCCLM3 and HepG2 cells by the transfection of pcDNA3.1/RAET1K. The transfection efficiency was confirmed by real-time PCR (n=5). Next, HCCLM3 and HepG2 cells were transfected with pcDNA3.1/RAET1K and examined for (B) miR-100-5p expression by real-time PCR (n=5); (C-D) the glucose uptake and lactate concentration. (n=3) ***P*<0.01.
