# Supplementary figures and images for: HIF1A activates the transcription of lncRNA RAET1K to modulate hypoxia-induced glycolysis in hepatocellular carcinoma cells via miR-100-5p
Source: Cell Death Dis. 2020 Mar 9;11(3):176. doi: 10.1038/s41419-020-2366-7 (PMC7062743; doi:10.1038/s41419-020-2366-7)

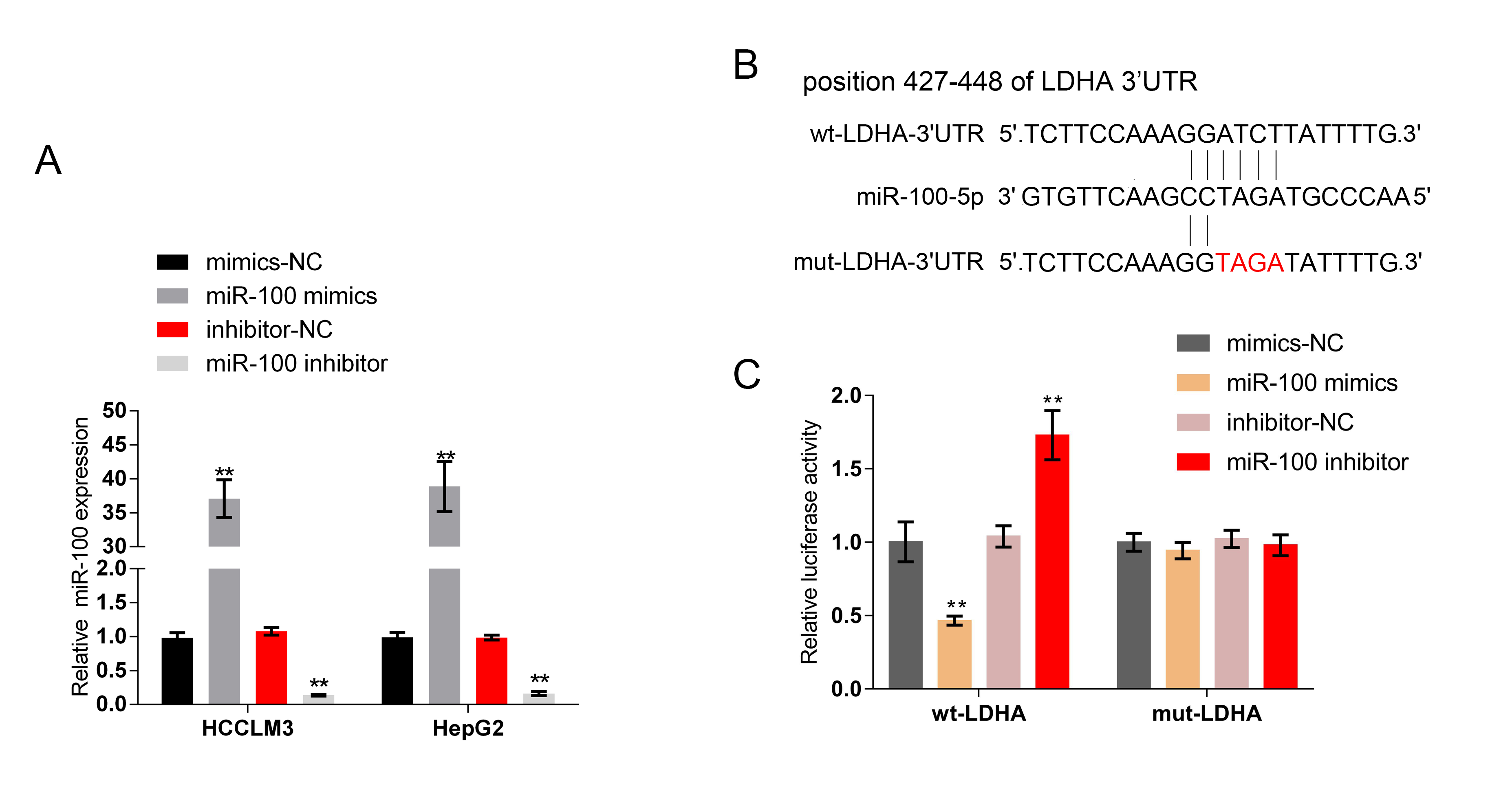

Supplement: Supplementary file 2 — fig.s1 [file 41419_2020_2366_MOESM2_ESM.tif]

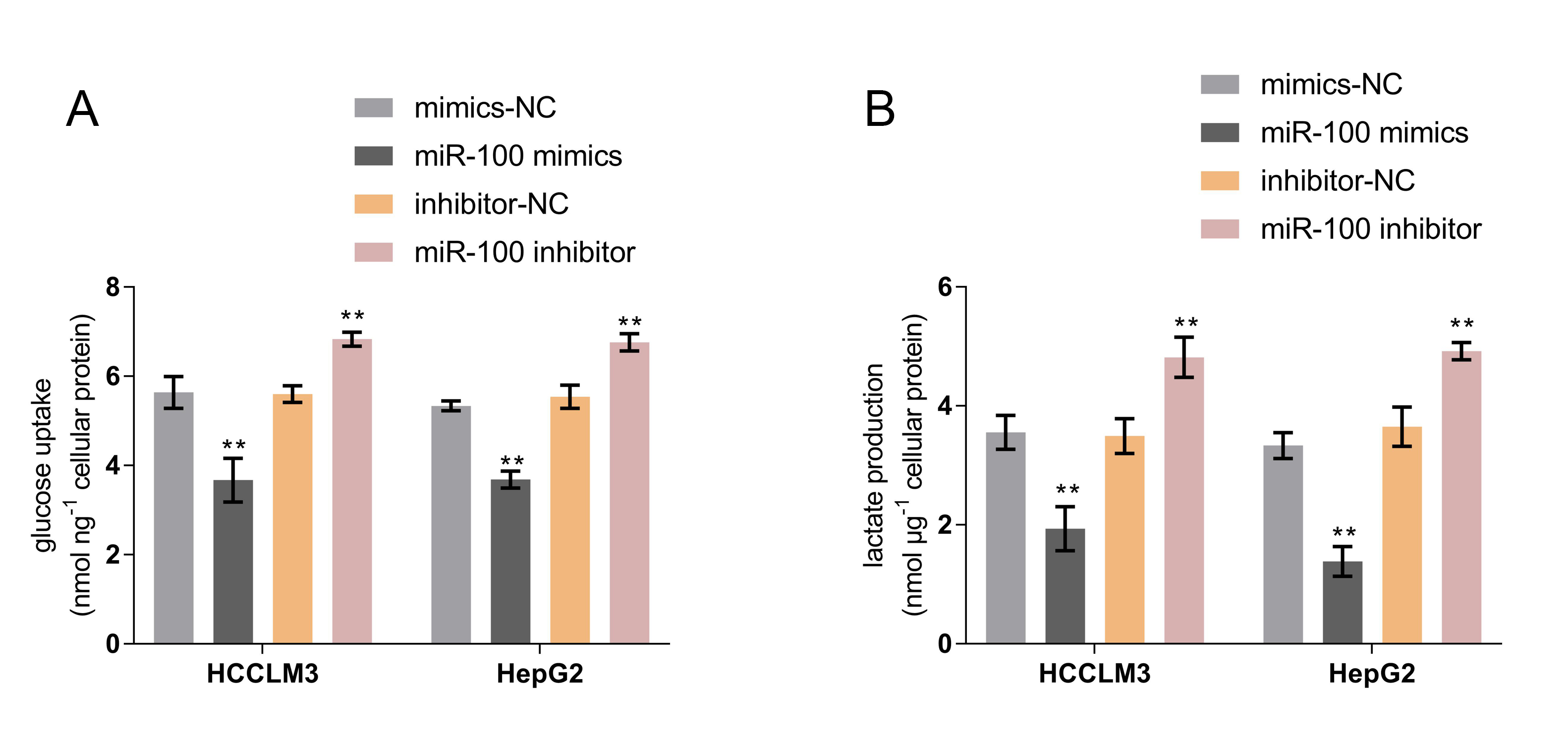

Supplement: Supplementary file 3 — fig.s2 [file 41419_2020_2366_MOESM3_ESM.tif]

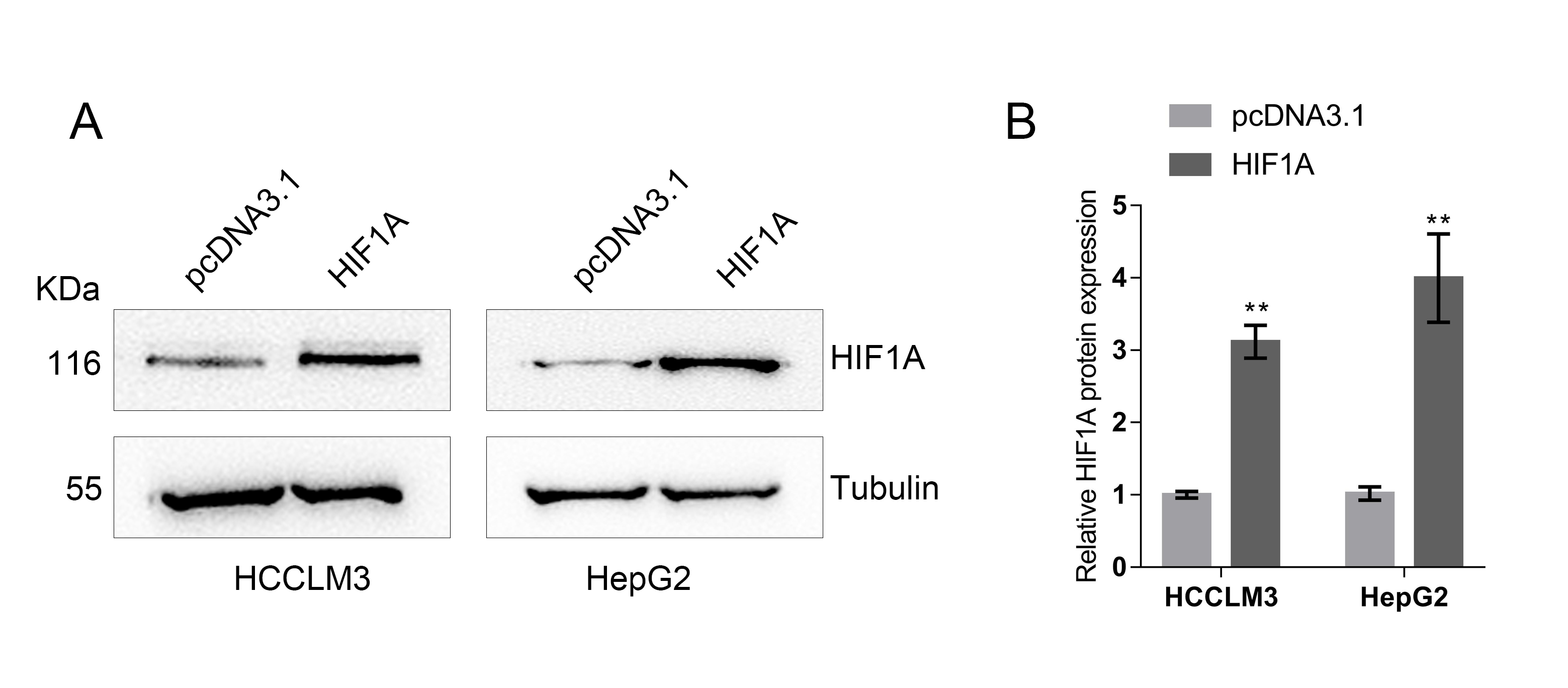

Supplement: Supplementary file 4 — fig.s3 [file 41419_2020_2366_MOESM4_ESM.tif]

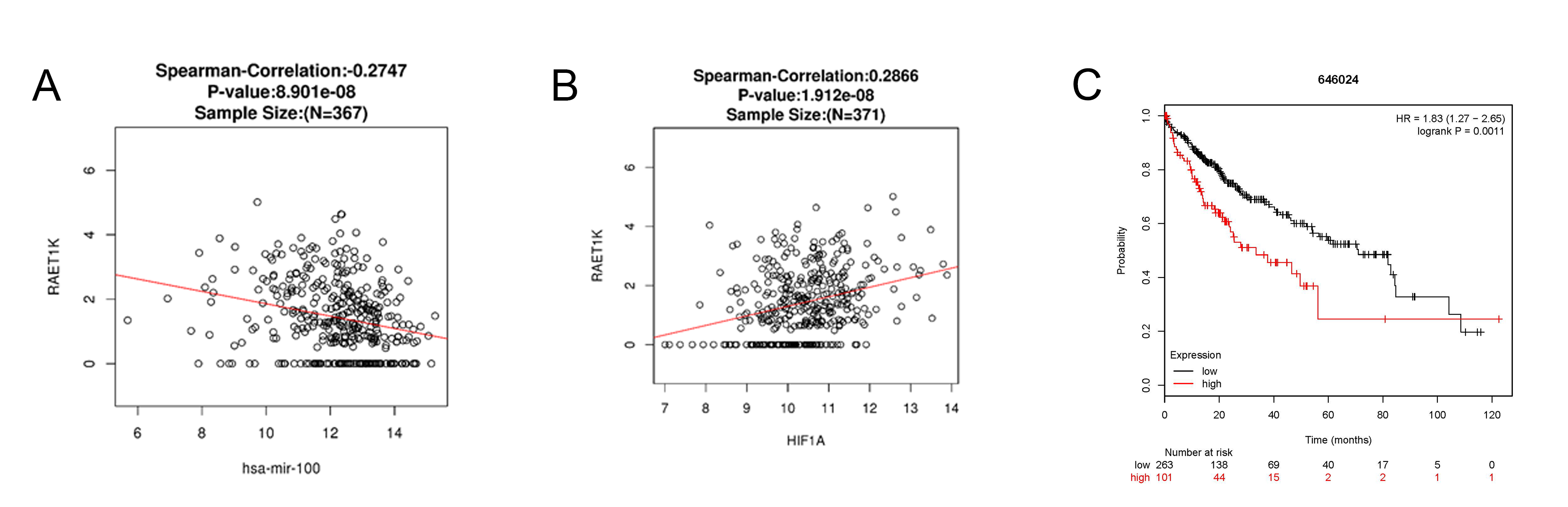

Supplement: Supplementary file 5 — fig.s4 [file 41419_2020_2366_MOESM5_ESM.tif]

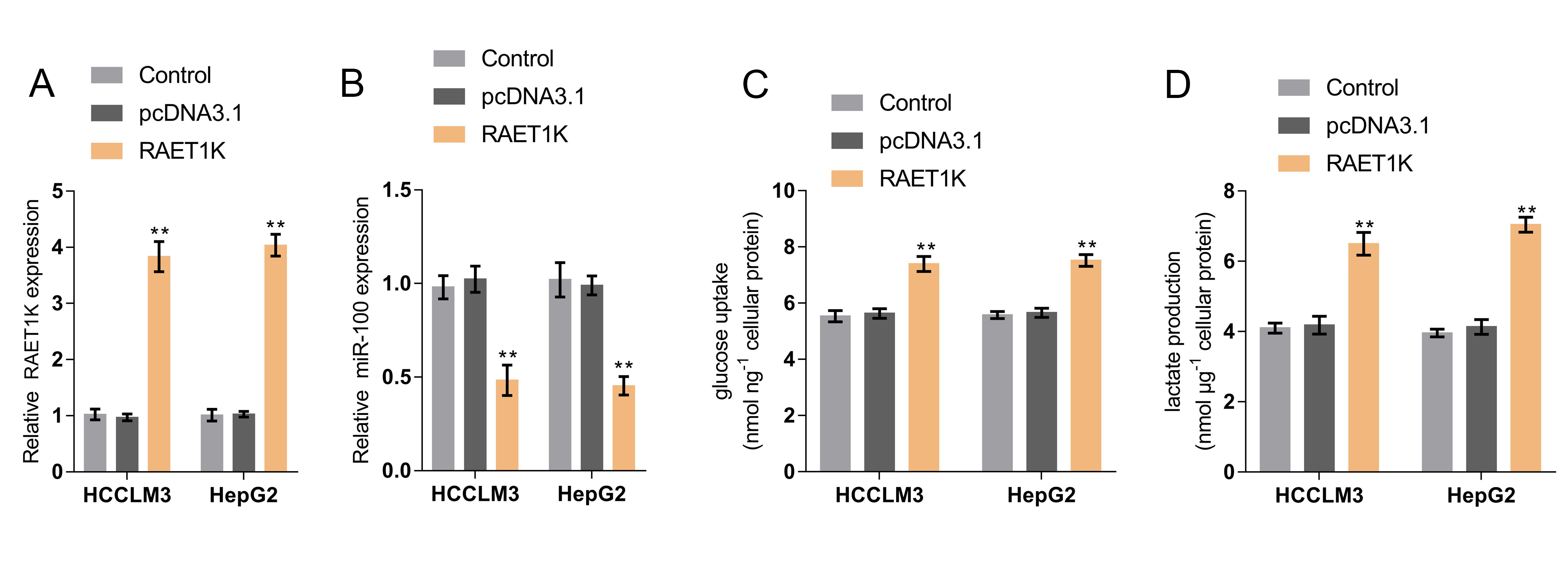

Supplement: Supplementary file 6 — fig.s5 [file 41419_2020_2366_MOESM6_ESM.tif]
