# Supplementary material for: HIF1A activates the transcription of lncRNA RAET1K to modulate hypoxia-induced glycolysis in hepatocellular carcinoma cells via miR-100-5p
Source: Cell Death Dis. 2020 Mar 9;11(3):176. doi: 10.1038/s41419-020-2366-7 (PMC7062743; doi:10.1038/s41419-020-2366-7)
Supplement: Supplementary file 7 — table s1 [file 41419_2020_2366_MOESM7_ESM.docx]

Table S1 primer sequence

| Name | Forward/sense 5’-3’ | Reverse/antisense 5’-3’ |
| --- | --- | --- |
| Si-RAET1K#1 (1840-1862) | GCCUAGAUUUAUAGUUAAATT | UUUAACUAUAAAUCUAGGCTT |
| Si-RAET1K#2 (297-319) | CACACUAAGACAAACUGAATT | UUCAGUUUGUCUUAGUGUGTT |
| Si-RAET1K#3(1853-1875) | GUUAAAUAAAGAUGUCUAUTT | AUAGACAUCUUUAUUUAACTT |
| RAET1K overexpression | CCGGAATTCCTTGCCCCGCGTTCC | CCGCTCGAGTTTTTTTTTTCTTTGAAAATAGACA |
| Si-HIF1A#1(1418-1140) | GCUAUUCACCAAAGUUGAATT | UUCAACUUUGGUGAAUAGCTT |
| si-HIF1A#2 (2271-2293) | CCAUAUAGAGAUACUCAAATT | UUUGAGUAUCUCUAUAUGGTT |
| Si-NC | UUC UCC GAA CGU GUC ACG UTT | ACG UGA CAC GUU CGG AGA ATT |
| HIF1A overexpression | AAATATGCGGCCGCTCAGTTAACTTGATCCAAAGCTCTG | AAATATGCGGCCGCATGAGCTCCCAATGTCGGAGT |
| Mimics NC | UUCUCCGAACGUGUCACGUTT | ACGUGACACGUUCGGAGAATT |
| Inhibitor NC | CAGUACUUUUGUGUAGUACAA |  |
| MiR-100 mimics | AACCCGUAGAUCCGAACUUGUG | CAAGUUCGGAUCUACGGGUUUU |
| MiR-100 inhibitor | CACAAGUUCGGAUCUACGGGUU |  |
| RT-PCR RAET1K | TTAGAGTGACAGGTGGAAGGTGATA | CCAGAAAGGCACAGTGGTGAGTA |
| RT-PCR Tubulin | TGGACTCTGTTCGCTCAGGT | TGCCTCCTTCCGTACCACAT |
| RT-PCR miR-100 | RT:GTCGTATCCAGTGCGTGTCGTGGAGTCGGCAATTGCACTGGATACGACCACAAG  F:GAACCCGTAGATCCGAA | CAGTGCGTGTCGTGGA |
| RT-PCR U6 | CTCGCTTCGGCAGCACA | AACGCTTCACGAATTTGCGT |
| CHIP-RAET1K | CAGGGCGGGGTGGAGCAT | AGTCGGGGTCGAGGCGGATA |
| CHIP-VEGF | GGGCACTGGCTGAGTCCTTA | GTGTTTCCCTCTGGGGCTTT |
| miR-100 probe | CACAAGTTCGGATCTACGGGTT |  |
| RAET1K probe | CCAGAAAGGCACAGTGGTGAGTA |  |
